# A novel positioner for accurately sitting the acetabular component: a retrospective comparative study

**DOI:** 10.1186/s13018-019-1331-6

**Published:** 2019-08-28

**Authors:** Liangliang Cao, Yuehui Wang, Shiping Zou, Hui Cheng

**Affiliations:** Department of Joint Surgery, Zhengzhou Orthopaedic Hospital, 58 Longhai Middle Road, Zhengzhou City, Henan Province China

**Keywords:** Positioner, Rotation center, 3D printing, Total hip arthroplasty, Computer-assisted

## Abstract

**Introduction:**

In this study, we described a positioner which allows a combination of preoperative plan and intraoperative insertion of the cup to improve the reconstruction of the rotation center of the hip.

**Materials and methods:**

A retrospective study was conducted on 32 consecutive patients (group A) using this positioner and 40 consecutive patients (group B) using conventional method; radiological parameters and clinical measurements before operation and at last follow-up were collected and evaluated.

**Results:**

Group A had a reconstructed center of rotation (COR) that was 0.19 mm closer to the anatomic COR in height (*P* < 0.005), compared with group B with 3.45 mm vertical dislocation. There were no statistically significant differences in the horizontal displacement between the two groups. The accuracy of cup inclination was 42.14 ± 3.57 in the group A and 38.73 ± 7.65 in the group B (*P* = 0.015). The accuracy of cup anteversion was 14.82 ± 1.44 in the group A and 13.08 ± 5.95 in the group B (*P* = 0.082). All cups in the group A were radiologically stable, while one cup in the group B was radiologically unstable and was successfully treated with second-stage revision. Both of the groups obtained a higher mean postoperative Harris Hip Score.

**Conclusions:**

Utilizing this positioner helps to restore the COR position more precisely and provides satisfactory radiological and clinical outcomes in the short term, and more studies are required before its widespread adoption for complicated cases.

The total hip arthroplasty (THA) has been one of the most effective and successful treatments of hip disorders in terminal state [1, 2]. Reconstructing the optimal biomechanics, especially for the rotation center of the hip (RCH), to get the better longevity of implant and clinical outcomes has been received more and more attention [3, 4]. Although numerous advances in technique and prostheses have been achieved, investigators have addressed the prevalence and etiology of dislocation after primary THA in most previous clinical studies [5, 6]. The position of RCH, which depends on the position of acetabular cup, influences abductor muscle function, soft tissue balancing, joint reactive forces, range of motion, liner wear rate, implant stability, gait, and, consequently, patient satisfaction and clinical outcomes [7, 8]. However, RCH is not a definite anatomical structure, and its position rests with variables of acetabular cup depth, height, and angular position (anteversion and inclination) [9, 10]. These variables are able to be obtained on the postoperative X-ray of CT via many kinds of ways, but the methods that help to definite the position of RCH during surgery are seldom, mainly relying on experience of the surgeon, which attributes to great differences of accuracy rate of RCH between different medical centers [11–13]. So a simple, reproducible, and affordable method is needed to resolve the problem. This study introduces a RCH positioner based on preoperative CT model and 3D printing to assist in locating the hip joint center during operation.

## Patients and methods

### Patient selection

This retrospective study was conducted with the approval of the Ethics Committee of our institution, and all patients were informed and provided consent preoperatively. A total of 72 hips from 72 patients with unilateral hip lesion and without pelvis and spine malformation who underwent primary THA for osteoarthritis, femoral neck fracture, and osteonecrosis of the femoral head at our institution between March 2015 and July 2017 were included. One single reconstructive surgeon at our medical institutions completed these operations. We examined and regarded 32 consecutive hips in 32 patients (22 females and 10 males) who utilized this new technique and who were diagnosed with osteoarthritis (*n* = 2), femoral neck fracture (*n* = 12), or osteonecrosis of the femoral head (*n* = 18) as group A, and the others who utilized conventional ways to determine RCH as group B (*n* = 40, 28 females and 12 males) and who were diagnosed with osteoarthritis (*n* = 3), femoral neck fracture (*n* = 15), or osteonecrosis of the femoral head (*n* = 22). The average age of group A at the time of surgery was 66.8 years (range 32–87), and the average follow-up period was 1.8 years (range 1–2.6 years). The average age of group B at the time of surgery was 67.3 years (range 35–85), and the average follow-up period was 2.2 years (range 1.2–3.4 years).

### Design of the positioner

**Fig. 1:**
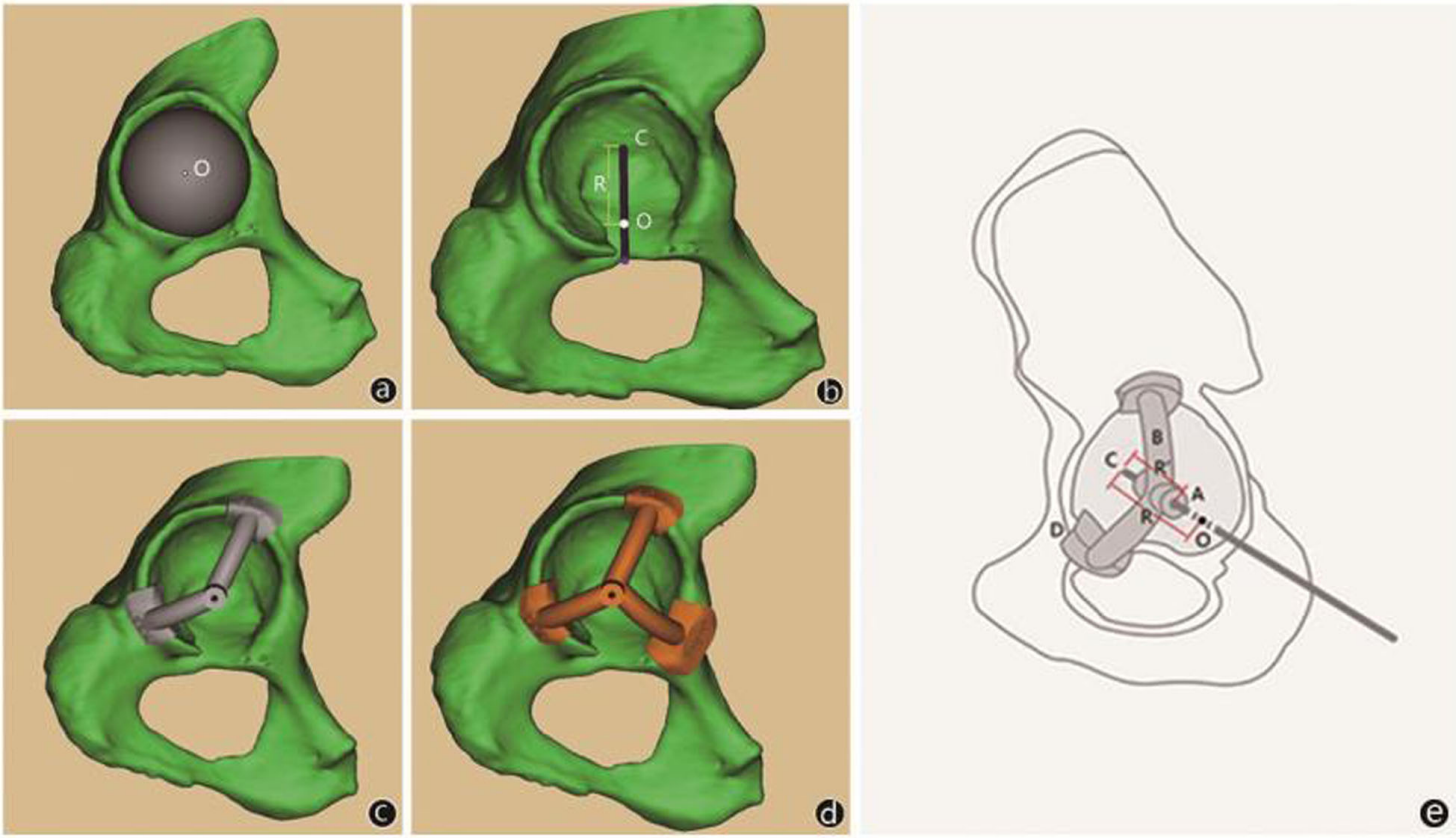
The best-fit sphere model implanted in the true acetabulum in Mimics. **a** Center (O), radius (R), reaming center (C), and the positioning line (H). **b** The positioner with two and three brackets (**c**, **d**)**.** Diagrammatic drawing of the positioner and relevant parameter (**e**)

**Fig. 2:**
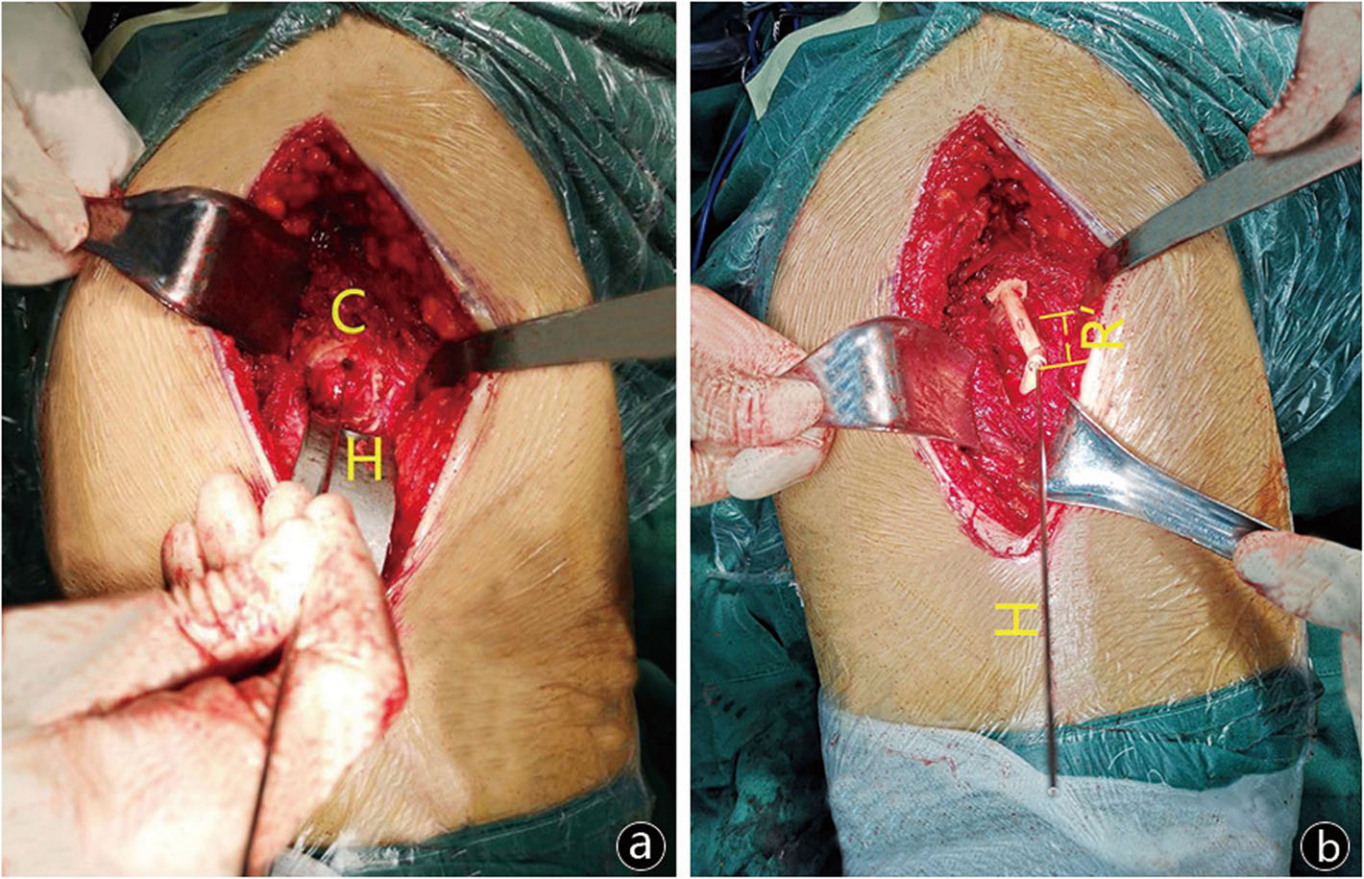
C is the intraoperative grinding center, and H is the direction of Kirchner wire, that is to say, the direction of grinding (**a**). R’ is the depth between the lateral border of cylindrical center hole and C (**b**)

All 3D models of the acetabulums and positioners were established by one single technician, which were based on the data obtained from computed tomography scans at 1-mm intervals and then imported into Mimics. And when establishing the positioner, the acetabulum is regarded as part of the sphere; then, fit the different size of the diameter of spheres with the acetabulum and chose the sphere with suitable diameter and the maximum ground contact with acetabulum to obtain the center of the sphere (O) and sphere diameter (R). According to the inclination values of 40° and anteversion values of 15° [14], determine a positioning line(H) through O and R, and the intersection of the H and acetabulum bottom was the intraoperative grinding center (C). The positioner was composed of three components: cylindrical center hole (A), two or three arc brackets (B) based on the condition of ossification of acetabulum edge, and clamp groove (D). The direction of the cylinder-shaped hole needed to be consistent with the direction of the positioning line (H) to determine the direction of intraoperative Kirchner wire, and at the same time, the distance (R’) from the lateral border of A to C needed to be recorded to determine the grinding depth (Figs. 1 and 2).

### Surgical technique

All operations were conducted through the Hardinge approach by a single trained surgeon. All the acetabulums were reamed sequentially into an approximately hemispherical shape until reaching viable host bone.

For the traditional technique, transverse acetabular ligament and cotyloid fossa were the common reference for investigating the location of intraoperative grinding center. While for reaming orientation and depth, surgical experience was always the main reference, which was especially significant for reaming concentrically with different sizes to ensure acetabular steady.

For the new technique, capsule flaps were needed to be removed to the host bone during operation, according to the relative locations of clamp groove and acetabulum, and then fix the positioner on the edge of acetabulum. After that, a Kirchner wire with scale was pulled through the cylindrical hole, and C and reaming direction (H) were obtained. Then, take out the positioner and K-wire, and the acetabulum was reamed sequentially into an approximately hemispherical shape. In the interval of changing reaming size, the orientation and depth were reconfirmed with installing positioner and K-wire to avoid intuitive error until the predesigned scale on K-wire reached to A.

### Radiographic preparation

All the radiographs were obtained by well-trained and experienced technicians in a standardized manner as follow: patients were supine on the photography table, with median sagittal plane coincided with the cassette midline, and the lower limbs were fully extended and were placed in internal rotation about 15°. The X-ray projection was centered over the point 3 cm below the pubic symphysis midpoint with a beam-patient distance of 100 cm, and the beam was vertically injected into the cassette. Only standard bilateral hip anteroposterior radiographs were gathered, including isometrical bilateral obturator foramens, the tip of coccyx locating at the level and in the center of the pubic symphysis, and longitudinal axes of bilateral femurs parallel to each other and to the longitudinal central axis of the pelvis (Fig. 2).

### Radiographic measurements

**Fig. 3:**
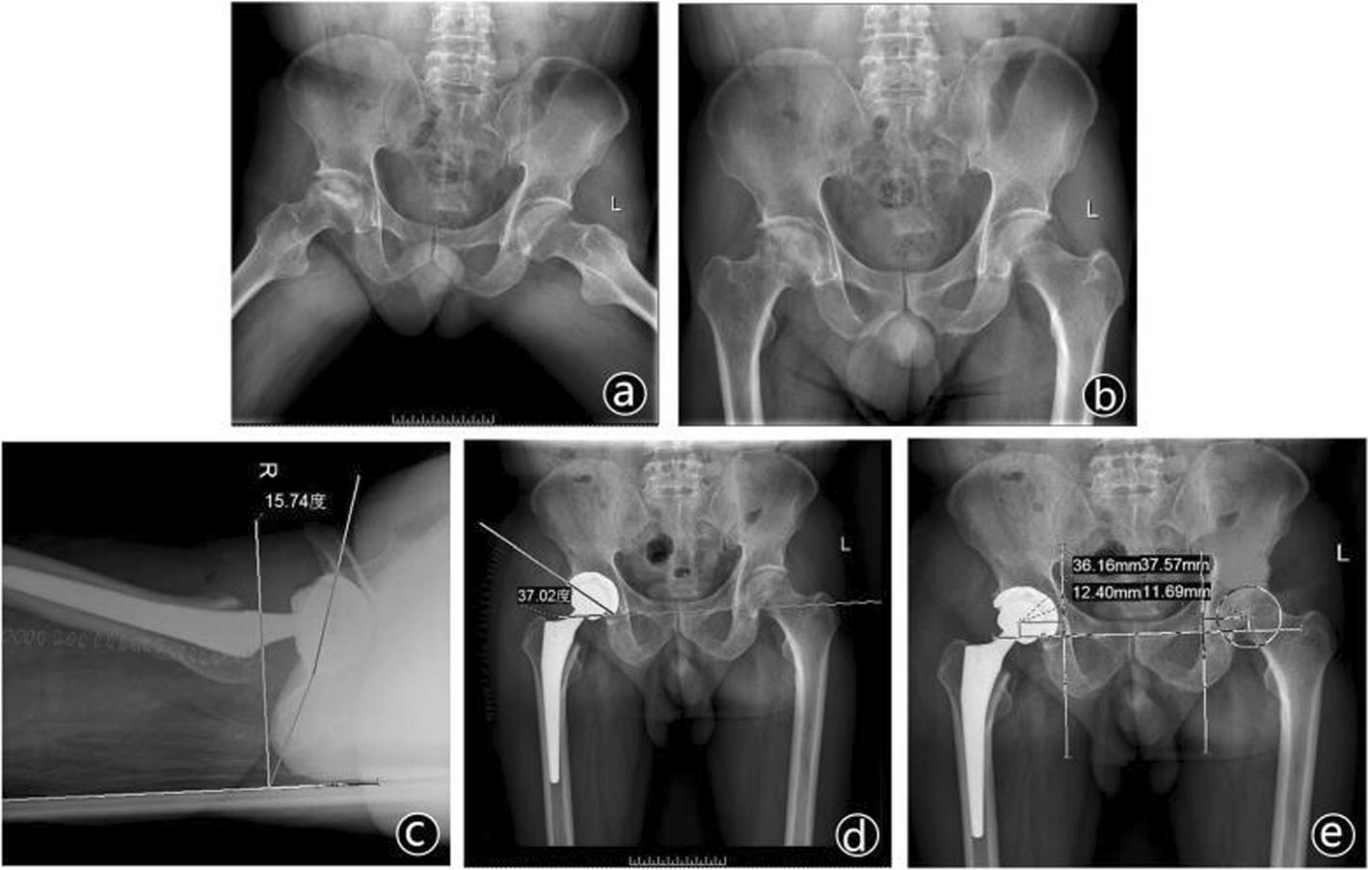
A preoperative radiograph showing osteonecrosis of the right femoral head (**a**, **b**). The anteversion angle, inclination angle, and vertical and horizontal displacement on the follow-up radiograph taken 1 year after right THA (**c**, **b**, **d**)

The radiographic measurements were analyzed on a consensus basis by three authors who did not perform the procedures using AP pelvic radiographs from the patient’s initial postoperative visits (typically between 2 and 6 weeks). The differences of measurements, including vertical RCH displacement, horizontal RCH displacement, anteversion values, and inclination values between the operative side and non-surgery side were assessed. In this study, the center of the prosthetic femoral head and femoral head of the unaffected side were determined to represent the reconstructed RCH and anatomical RCH. We measured the positions of the reconstructed RCH referring to the ipsilateral teardrop and the unaffected side RCH as the absolute and relative positions respectively. An interteardrop line was drawn connecting the most inferior borders of the two pelvic teardrops. All vertical absolute measurements of both sides were made from the interteardrop line. All horizontal absolute measurements were made from a line perpendicular to the interteardrop line, which passed through the center of the teardrop. The relative positions were determined as the vertical and horizontal distances from the reconstructed RCH to non-surgery RCH. Then, set up a coordinate system with O1 as the origin, denoting RCH of non-surgery side, and obtain the relative values by subtracting the non-surgery side values from postoperative ones. The circle with a center of O1 and radius of 5 mm was made as the “target zone.” The anteversion angle and inclination angle were the angle between the acetabular cup edge and interteardrop line and the angle between the vertical line of the coronal plane and the acetabular cup edge respectively (Fig. 3).

In the clinical assessment, the Harris score was assessed preoperatively at the final follow-up. The degree of postoperative improvements was classified according to this method as a very good improvement, good improvement, fair improvement, and failure. Radiological failure was defined if the acetabular construct was evaluated as unstable [15]. Postoperative complications and reoperations were also recorded at each follow-up. Clinical failure was defined as the demand for further acetabular revision for any reason [16].

### Statistical analysis

The proportion of patients who meet the re-construction criteria was calculated. Comparisons were performed using chi-square tests for categorical, Mann-Whitney *U* test and *t* tests for continuous variables. A *P* value of < 0.05 was set as the threshold to determine statistical significance for the results. All statistical analyses were completed using SPSS version 21 (IBM Corp., Armonk, NY, USA).

## Results

There was no significant statistical difference in patient demographics and baseline characteristics between the two groups (Table 1). The mean Harris Hip Score of the two groups were both improved at the final follow-up, but we found no significant difference between the two groups. One case assessed as showing a fair improvement in group B had dislocation of prosthesis that occurred 6 months after surgery, and the patient was successfully treated with manual reduction. No other cases underwent additional surgical treatments in the follow-up period.

**Table 1:** Patient demographics and baseline characteristics

	Group A (32 hips, 32 patients)	Group B (40 hips, 40 patients)	*P* value
Demographic characteristics			
Age* (year)	66.8	67.3	0.856*
Female/male	22/10	28/12	0.909^#^
Height* (cm)	168.4 ± 6.5	167.1 ± 8.3	0.471*
Weight* (kg)	73.5 ± 8.7	71.8 ± 9.4	0.433*
Body mass index* (kg/m^2^)	26.4 ± 3.6	27.2 ± 2.5	0.271*
Osteoarthritis/femoral neck	2/12/18	3/15/22	1.000^#^
Fracture/spontaneous osteonecrosis			
Operative status			
Estimated blood loss* (ml)	334.8 ± 22.9	322.3 ± 30.7	0.060*
Harris score	13/19/0/0	15/24/1/0	0.685^&^
(Excellent/good/fair/poor)			
Duration of surgery* (min)	125.6 ± 13.7	132.2 ± 20.2	0.119*

*The *P* values were determined with the *t* test

^#^The *P* values were determined with the chi square test

^&^The *P* values were determined with the Mann-Whitney *U* test

**Fig. 4:**
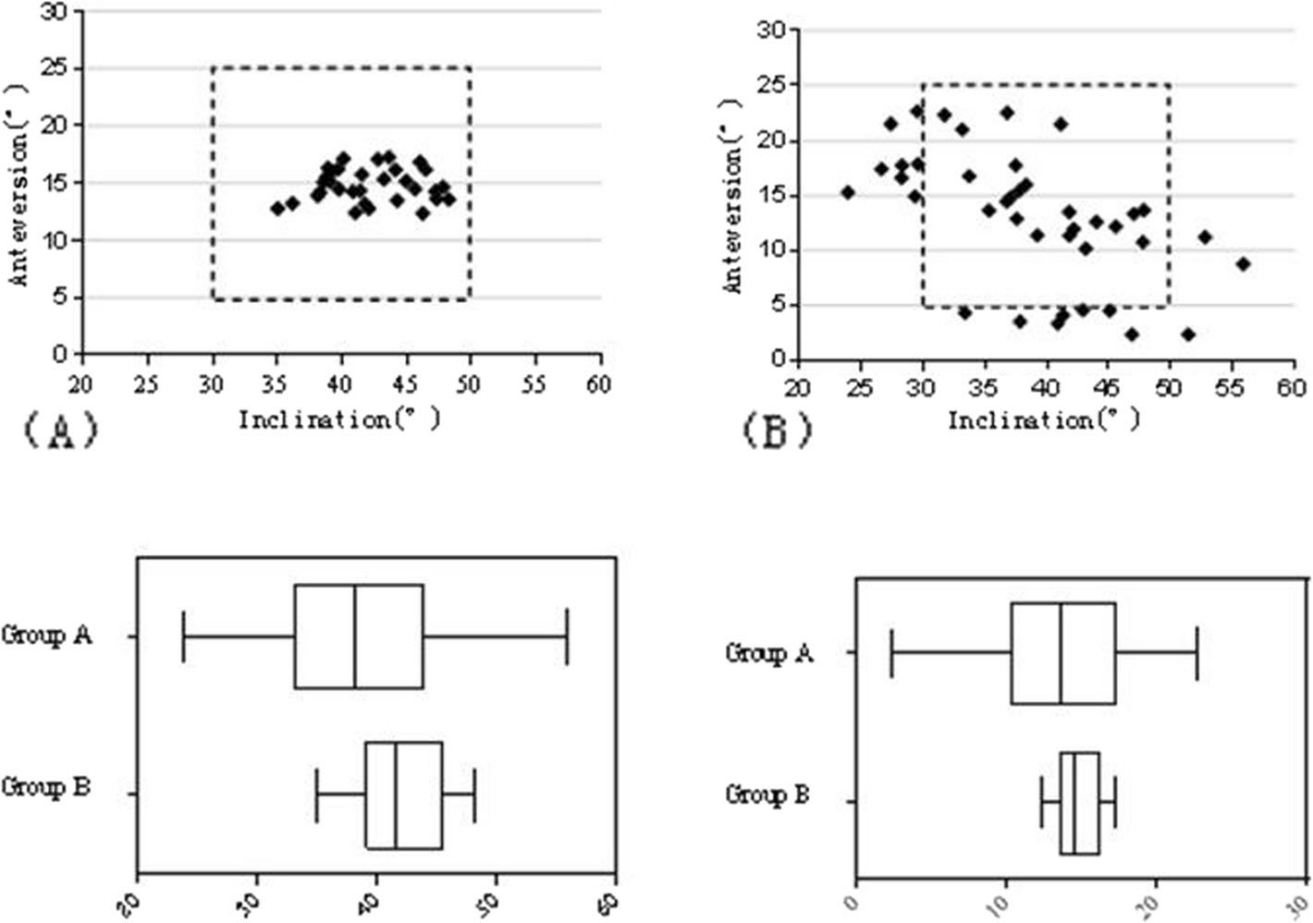
The distribution of the cup placement angles in group A (**a**) and Group B (**b**),the dotted lines indicate the Lewinnek’s safe zone. Box whisker graph comparing the inclination and antevertion angular setting respectively of cups in the two groups

**Fig. 5:**
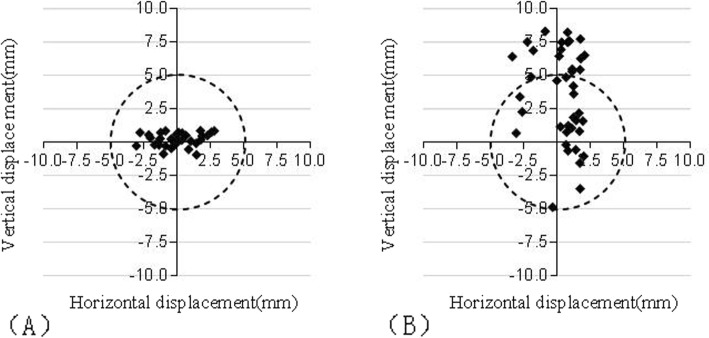
Displayed is a scattergramst discribing the vertical and horizontal displacement of the reconstructed RCH relative to native RCH in two groups (**a**, **b**). Dotted circles indicate the range of the “target zone”

In the radiographical assessments, according to the standards of RCH reconstruction defined by Lewinnek, the anteversion and inclination of all cases in the group A and 22 of the 40 cases (55%) in the group B fell within the dotted lines (Fig. 4). The inclination angle and anteversion angle of the cups in group A were 42.14° ± 3.57° and 14.82° ± 1.44° respectively, while those in group B were 38.73° ± 7.65° and 13.08° ± 5.95° respectively (*P* > 0.05) (Table 2). The scatter of vertical and horizontal dislocation deviated from the targeted zone by 5 mm is shown in Fig. 5. The mean vertical dislocation of the RCH in group A and group B was 0.19 mm (range − 0.88–0.86 mm, SD 0.50 mm) and 3.45 mm (range − 4.88–8.30 mm, SD 3.46 mm) respectively. The difference was statistically significant (*P*< 0.05). But there was no statistically significant difference in horizontal dislocation between the two groups, which was − 0.04 mm (range − 3.05–2.29 mm, SD 1.55 mm) and 0.43 mm (range − 3.34–2.06 mm, SD 1.54 mm) respectively. All cases in group A and 24 of the 40 cases (60%) in the group B fell within the target zone (Table 3).

**Table 2:** Radiological measurements

	Vertical displacement of RHC*	Horizontal displacement of RHC*	Inclination (°)^#^	Anteversion (°)^#^
Group A	0.19 ± 0.5	− 0.04 ± 1.55	42.14 ± 3.57	14.82 ± 1.44
Group B	3.45 ± 3.46	0.43 ± 1.54	38.73 ± 7.65	13.08 ± 5.95
*P* value	0.000^#^	0.130^#^	0.015*	0.082*

^#^The *P* values were determined with the Mann-Whitney *U* test

*The *P* values were determined with the *t* test

**Table 3:** Comparison of accuracy in acetabular cup placement

	Safe zone of cup position	Safe zone for inclination	Safe zone for anteversion	Safe zone for angle
Group A	32 (32)	32 (32)	32 (32)	32 (100%)
Group B	24 (40)	29 (72.5%)	32 (80%)	22 (55%)
*P* value	0.000	0.004	0.021	0.000

The *P* values were determined with the chi-square test

## Discussion

The position of COR following THA is an essential factor that affects the clinical outcomes and longevity of prosthesis [17]. Malposition of RCH may eventually lead to pseudosubluxation [18]. Superior or lateral placement of the cup as a risk factor may result in aseptic loosening of the implants, discrepancy of the leg, bony impingement, and decreased abductor muscle tension [19–21]. In addition, an increase of more than 45° in the abduction angle has been described as a risk factor for increase in linear and volumetric wear of the prosthesis, and decrease of this angle may lead to restriction of abduction, and even subsequent mechanical failure [21, 22]. Several methods to determine the RCH have been described in the literature to help correct cup placement. Doyle et al. [23] described a surface-mounted device involving a frame, laser unit, and mirror to improve the accuracy of abduction angle of the cup. This method helped to get an accurate abduction angle, but was unhelpful in determining anteversion angle and position of intraoperative grinding point. Archbold et al. [24] and Idrissi et al. [25] hold the opinion that transverse acetabular ligament is a reference to the position and anteversion angle of the cup, which is used as traditional method currently. However, acetabular reamers should be done carefully to avoid excessive reaming that may potentially result in transforming a grade 3 ligament into a grade 4 by destroying it. And to restore the anatomic RCH in this way, the ligament should embrace the final acetabular reamer, which lies on the operator’s intuitive judgment, and would absolutely cause deviation. Ha et al. [26] suggested that the transverse acetabular notch and anterior acetabular notch—a notch at the anterior acetabular margin, on acetabulum rim—could be presumed as a landmark for determination of intraoperative abduction and anteversion. But this method needs to remove transverse acetabular ligament, which is helpful for insertion and stability of cup [27].

Despite advances in surgery technique, the acetabular reaming depth—usually 2 mm—for anatomic restoration of RCH and the abduction angle of the acetabular component cannot be judged on the bony landmarks of the native acetabulum [28]. If the reaming depth is too deep or shallow, the position of RCH will be affected obviously in horizon direction, which may lead to a revision surgery. Considering the hemispherical character of the cup, the native acetabulum is subhemispherical, which inadvertently leads to displacement of RCH when the acetabular component is fully implanted [9, 10]. Besides, all these methods require rather intuitive judgment on the position, orientation, and depth of reaming. Several studies reported the significant differences in the radiographic and clinical outcomes between surgeons with different levels of surgical expertise, such as change in the COR, initial cup position, cup orientation, number of cups within the safe zones, and dislocation rate. The percentage of hips located within the safe zones varies from 70.5 to 25.7% [11–13, 17]. In the present study, patients using this positioner have less vertical displacement of the COR of the hip when compared with those in group B. In addition, the inclination and anteversion angles in group A are more stable and all in the safe zone, portion of which is significantly higher than that of group B—only 55%. These results just imply the hip surgeons’ fear of wall defect resulting from reaming, leading to a relatively higher RCH and smaller inclination angles as this study revealed. In addition to intuitive bias, the position on the operating table, the dislocation of the native hip, and the use of retractors may alter the pelvis and, thus, the acetabular version [29]. Although armed with a universal standard of orientation, as suggested by Lewinnek et al. [14], surgical experts may always be confused about the cup orientation which is intraoperatively perfect but is out of the safe zone on the postoperative X-ray. Although navigation technology has obvious advantages in precise insertion of the acetabular component, the surgical experts may be discouraged by the additional cost and increased duration of surgery [30, 31]. In order to achieve an acetabular accurate position and orientation for the acetabular cup, there is a need for technical aids and tools for orthopedists, especially in primary hospitals.

In this study, we introduce a simple and reproducible method which has been proved to be effective. When performing a preoperative planning in Mimics, we need to get a ball highly uniform with the acetabulum on radius and then design the locator, according to the position, radius and standard of abduction and anteversion angle. The locator was printed by 3D printing technology for intraoperative positioning of the rotation center, which can effectively realize the combination of preoperative planning and intraoperative positioning. When reconstructing the rotation center of the hip joint, the direction of reaming, namely, the anteversion angle and abduction angle, uniqueness of which is guaranteed by cylindrical center hole (A), the concentric center of reaming, and the depth of reaming—usually 2 mm—need to be taken into full consideration [28]. After that, the rotation center is determined. The locator is simple and easy to realize the unification of them, regardless of the intraoperative positioning of the patient. In addition, this positioner could be made as the specific anatomic characteristic to avoid the anatomic and intuitive deviation, and results in this study are consistent with it. The abduction angle is smaller and even out of the “safe zone,” which may be resulted from fear of acetabular protrusion, while the values in group A range around 45° as planned preoperatively. Besides, this method could enhance operative confidence without having to consider the patient position, deviation from their poor experience.

In this study, we have developed a positioner to improve rotation center in a small clinical trial, and the results have recognized the superiority of this method. However, due to the limitation of the positioner, the positioning device and the Kirchner wire need to be taken out after determining the reaming direction, and then the acetabulum was reamed according to the direction of Kirchner wire, resulting in a lack of precision. The combination of the positioner and the ream cannot be realized yet in this study. Only vertical and horizontal displacement was considered, and only radiographic appearance was assessed in this analysis. In addition, the follow-up time was relatively short, so the effect on biomechanics and implant fixation was still needed for the determination of postoperative clinical efficacy and prosthesis wear rate, and further studies are needed for the cases with severe acetabular structure damage and variation.

## Conclusion

Postoperative functional and radiological improvements show that utilizing this positioner helps to restore the COR position more precisely and provides satisfactory radiological and clinical outcomes in the short-term, and provide a new option for reconstruction of hip rotation center.

## Data Availability

We declare that the materials described in the manuscript will be freely available to all scientists for non-commercial purposes.

## Abbreviations

COR: Reconstructed center of rotation
RCH: Rotation center of the hip
THA: Total hip arthroplasty
